# Human Umbilical Cord Wharton Jelly-Derived Adult Mesenchymal Stem Cells, in Biohybrid Scaffolds, for Experimental Skin Regeneration

**DOI:** 10.1155/2017/1472642

**Published:** 2017-12-31

**Authors:** Pia Montanucci, Camilla di Pasquali, Ivana Ferri, Teresa Pescara, Ilaria Pennoni, Paola Siccu, Angelo Sidoni, Valerio Cervelli, Giuseppe Basta, Riccardo Calafiore

**Affiliations:** ^1^Department of Medicine, Section of Cardiovascular, Endocrine and Metabolic Clinical Physiology and Laboratory for Endocrine Cell Transplants and Biohybrid Organs, University of Perugia, Piazza Lucio Severi, Perugia, Italy; ^2^Department of Plastic and Reconstructive Surgery, University of Rome “Tor Vergata”, Via Montpellier 1, 00133 Rome, Italy; ^3^Department of Experimental Medicine, Section of Pathologic Anatomy and Histology Medical School, University of Perugia, Perugia, Italy

## Abstract

The ultimate goal for skin tissue engineering is to regenerate skin lesions to allow the full restoration of morphological and functional properties as what they were before injury. To this end, we have assembled a new prototype of a biomimetic human umbilical cord adult mesenchymal stem cell (hUCMS)/fibrin-based scaffold. We have fully characterized the proposed dermal equivalent (DE) in vitro, to assess morphological, functional, and biological properties of the encased cells. We transplanted DE subcutaneously into immunocompetent rodents, to verify its full biocompatibility. Finally, we studied DE graft effects on full-thickness wounds, in immunocompetent mice to demonstrate its capability to drive the healing process in the absence of significant scarring tissue. The excellent outcome of these in vivo studies fuels hope that this new approach, based on a biohybrid DE, may be applied to the operative treatment of skin lesions (i.e., diabetic foot ulcers and burns) in man.

## 1. Introduction

Skin regeneration, despite steady progress, is filled with a number of unresolved issues. Autologous skin graft is the conventional treatment for wound repair, although it is burdened with several limits, from morbidity at the donor site to impossibility to treat large wounds resulting in poor esthetical results. The ultimate goal for skin tissue engineering is to regenerate skin to allow the complete structural and functional properties of the wounded area to go back to what they were before injury.

In this study, we aimed at developing a new regenerative biomimetic hUCMS/fibrin-based scaffold (DE). This dermal equivalent should be comprised of hUCMS and human fibrin. It is known that an optimum treatment for a wound regeneration, with no occurrence of unwanted scar, should include modulation of inflammation, induction of tissue's regeneration, mitigation of mechanical forces, and turnover and remodeling of ECM [1–3]. The purpose of the DE prototype proposed would be to meet these goals by providing a temporary coating and tissue protection in combination with stimulation of its growth.

Stem cells are a unique cell population hallmarked by self-renewal and cellular differentiation capability. These properties make them an attractive option for regenerative treatment of skin injuries and for esthetic procedures in plastic surgery. In particular, hUCMS (human umbilical cord Wharton jelly-derived mesenchymal stem cells) are adult stem cells, deemed able to differentiate, in vitro and in vivo, into several cell phenotypes [4–6]. hUCMS homing attitudes are likely related to the expression of receptors for chemokines and adhesion molecules [7]. Further clinical interest has been fueled by the observation that hUCMS are immunoprivileged, due to the lack of HLA-DR class II, while associated with immunomodulatory properties [8–10]. These features seem to relate to both humoral factors released from hUCMS (TGF-*β*1, IDO, iNOS, IL6, PGE2, HGF, VEGF) [11, 12] and their interactions with the target cells [13, 14]. In addition, hUCMS express all the three species of HLA, namely, HLA-E, HLA-F, and HLA-G families. These molecules are involved in the tolerogenic process occurring at the fetal-maternal interface [15]. In particular, it has been described that HLA-G released from human mesenchymal stem cells enables expansion of Treg cells [16]. We have recently shown immunoregulatory properties of hUCMS in two autoimmune disorders like type 1 diabetes mellitus (T1D) and Sjogren syndrome (SSJ) [10, 17]. hUCMS could help manage inflammation and stimulate the wound regeneration, with restoration of the entire skin components.

Fibrin is derived from human blood, and in view of future clinical applications, it is to be procured from the same subject to whom the DE is applied. Fibrin is a suitable molecule when it is intended for cell embodiment: in fact, it not only acts as a scaffold where the cells can be easily accommodated but also provides molecular signals to regulate cell function, since it contains binding sites for integrins, growth factors, and other ECM components including fibronectin and collagen. In vivo fibrin deposition may help the reinstatement of tissue homoeostasis and modulation of the inflammation [18]. Physically, the looming presence of DE in the wounded area is able to attenuate the mechanical tensile forces [19]. Indeed, there is a predisposition to form keloids and hypertrophic scars in areas under high stretching/contraction tension [20].

At last, innovation of this scaffold lies on its simple and reproducible assemblage process.

We aimed to explore the possibility of preparing a suitable DE, which may promote repair/regeneration of wall-thickness skin lesions, with minimal scar tissue and no infections or rejection, and create a low-inflammation wound environment. We have fully characterized the proposed bioscaffold as far as in vitro examination and in vivo studies in immunocompetent rodents are concerned.

## 2. Materials and Methods

### 2.1. hUCMS Isolation and Cell Culture

We isolated hUCMS from retrieved postpartum human umbilical cords as described by Montanucci et al. [4]. The isolated cells were seeded at a concentration of 6000–8000/cm^2^ nucleated cells per polystyrene cell culture flask, upon flask pretreatment with hyaluronic acid (HA) at 1 mg/ml flask, in CMRL Medium 1066 (Gibco, Invitrogen) supplemented with 10% fetal bovine serum, 2 mM L-glutamine, 100 U/ml penicillin/streptomycin, 50 *μ*g/ml amikacin, and 5 *μ*g/ml ciprofloxacin (normal medium (NM)) at 37°C and 5% CO_2_. The cells were maintained at 37°C in humidified 95% air. Cell expansion until 80% confluence was achieved by treatment with 0.05% trypsin/EDTA (Gibco) for 3′ at 37°C. Serum was subsequently added to block trypsin activity. Cells at IV–VIII culture passage were used to prepare fibrin scaffolds (S) and dermal equivalents (DE).

### 2.2. Preparation of a Fibrin Scaffold and Dermal Equivalents

Fibrin gel forming the scaffolds was prepared by mixing two solutions. One volume of plasma (obtained by centrifugation of healthy donors' fresh blood and filtered by 0.45 *μ*m pore filter) was added to 1.3 volumes of 0.9% sodium chloride with hUCMS. The latter were added to 0.9% NaCl in fibrin gel at a final concentration of 2.2 × 10^5^ cells/ml. Solution 2 was prepared by mixing twenty volumes of calcium chloride (1% CaCl_2_ in 0.9% NaCl) with one volume of tranexamic acid (100 mg/ml tranexamic acid in 0.9% NaCl). 1.8 ml of the cell/plasma mixture was poured into one of the 12-well culture plates. We obtained coagulation incubating cell/plasma solution for 2 minutes at 37°C in a humidified atmosphere. Then, normal medium was added to scaffolds. 18 hours after the production of fibrin scaffolds, DEs were assembled: 6 × 10^5^ hUCMS were seeded on the surface of each scaffold and cultured for 7/10 days in NM. The medium was changed every two days.

### 2.3. Histological and Immunohistochemical Analyses (IHC)

For histological and immunohistochemical analyses, DEs were fixed in 10% neutral buffered formalin for 24 h at room temperature, dehydrated, and paraffin-embedded. Paraffin-embedded specimens were cut with a rotary microtome (3.5 *μ*m thick slice) and stained with hematoxylin and eosin, Masson's trichrome, and cytokeratin staining with phloxine B-Orange G-Alcian Blue. Staining was performed according to standard histology protocols. Epidermis thickness was assessed by Photoshop software (Adobe) on microphotographs taken on 9 slides stained with H&E: they were equally distributed from the periphery to the center of the wound; within the wound area, three fields were selected and 5 thickness assessments were completed on each of them. Data were standardized on mean ± SD of normal epidermis (*z*-score). As for collagen evaluation, we analyzed the microphotographs of the slides upon Masson's trichrome staining by Photoshop software: we selected and counted, in the wound area, all pixels within the same color range of the adjacent normal skin, compatible with specific collagen staining. The pixel number was normalized for the area value where they were assessed. Pixels related to matched normal skin areas were also counted and normalized as said above. This analysis was carried out on 5 slides uniformly distributed from the periphery to the center of the wound per each sample. The obtained values, relative to the samples taken at the same experimental point (from two mice at 21 days and two mice at 36 days), have been calculated and normalized for normal skin area values. Data were presented as percent of detected collagen, as compared to that associated with normal skin.

Immunohistochemical analysis was done on 3.5 *μ*m slides using primary antibodies, specific for human Ki-67; smooth muscle actin (SMA); CK19; vimentin; E-cadherin; laminin; desmin; or mouse CD3, CD11b, or CD20. Automated immunohistochemical analysis was performed using the automated Leica BOND system (Leica Biosystems Newcastle Ltd., UK) on a Leica BOND-III instrument. The slides were counterstained with hematoxylin.

Cells on DE were compared with those maintained in the culture in NM. On this purpose, the cell block method was adopted. Cells cultured on flasks were treated with 0.05% trypsin/EDTA for 3 min at 37°C, whose activity was blocked by serum; washed with 0.9% NaCl; fixed in 10% neutral buffered formalin for 24 h, at room temperature; and treated for the following immunohistochemical analysis using primary antibodies specific for the same markers analyzed for DE. To count Ki-67-positive cells, 8 fields per cell block were chosen (40x magnification), 4-5 fields (20x magnification) for the internal scaffold and 5 for the surface of DE (40x magnification).

### 2.4. Immunofluorescence

For immunofluorescence analysis, we purged from paraffin and dehydrated the specimen sections. Then, antigen retrieval was performed by overnight incubation in Tris-EDTA (pH 9.0) buffered at 37°C in a humidified atmosphere. Permeabilization with 0.01% Triton X-100 in D-PBS for 10′ and block with 1% BSA in D-PBS for 45′ were performed. The primary antibody was added overnight at +4°C, while a secondary antibody was added for 1 h and 30′. Primary antibodies specific for human ku80 were used. Cell nuclei were counterstained with propidium iodide (Sigma) and finally slides were mounted with Mowiol 4-88 (Calbiochem), prepared according to the manufacturer's instructions, while 0.6% 1,4-diazabicyclo(2.2.2)octane (DABCO; Sigma) was used as an antifading agent. Images were collected on an Axio Observer A1 fluorescence microscope (Zeiss) equipped with an AxioVision software-driven camera.

### 2.5. Viability Assay

The CellTiter-Glo®3D Cell Viability Assay (Promega) and GloMax® Discover System (Promega) were used to evaluate cell viability; they were based on the quantization of the present ATP, which is a marker that evidences metabolically active cells.

### 2.6. Transmission Electron Microscopy (TEM) and Scanning Electron Microscopy (SEM)

Samples were prefixed in 2% glutaraldehyde; buffered with 0.2 M Na cacodylate (pH 7.4) for 2 h at 4°C; rinsed in the same buffer; postfixed with 2% osmium tetroxide, in the same buffer for 2 h; dehydrated in ethanol-graded series; and embedded in epon araldite. Ultrathin sections were stained with uranyl acetate and lead citrate and examined in TEM 400T Philips (B Philips) at 60 kV.

Samples were prefixed in 2% glutaraldehyde; buffered with 0.2 M Na cacodylate (pH 7.4) for 2 h at 4°C; rinsed in the same buffer; postfixed with 2% osmium tetroxide, in the same buffer for 2 h; dehydrated in ethanol-graded series; and critically point-dried and coated with gold palladium. Examination of the samples was conducted under SEM (Philips Scanning Electron Microscope, B Philips, The Netherlands) at 15 kV.

### 2.7. Transcriptional Expression Analysis by RT-PCR and qPCR

Total cellular RNA was extracted from the cultured cells using the TRI Reagent method (Bio-Rad Laboratories, Milano, Italy), phenol : chloroform extraction, and ethanol precipitations. cDNA was synthesized from 0.5–1 *μ*g total RNA using the iScript cDNA Synthesis Kit (Bio-Rad Laboratories). The obtained cDNA was used as a template in a PCR reaction, containing 400 nM of the primer pairs for amplification of the indicated gene product (Supplementary Table 1).

qPCR amplifications were performed by using the SsoFast EvaGreen Supermix as directed by the manufacturer (Bio-Rad Laboratories). The amplification was optimized for the MxPro 3000 Stratagene, and assays were run in triplicate, in a two-step PCR reaction under the following conditions: Taq polymerase activation at 95°C for 30^″^, followed by 40–45 cycles at 95°C for 10^″^, and annealing temperature at 60°C for 10^″^. PCR products were demonstrated to be a single PCR product by the melting curve and electrophoresis analysis. The relative quantification of mRNA of each gene was determined by the comparative 2^−ΔΔCt^ method where the target is normalized to the reference gene HPRT1. Statistical significance was determined by the calculation of the 95% CI [21].

### 2.8. Cytokine Assay

A mouse cytokine array for mIL1b, mIL6, and mIL10 (Aushon Ciraplex™) was purchased from Tema Ricerca Srl (Italy).

### 2.9. Animals

All immunocompetent CD1 mice were housed in the Perugia University Veterinary Service Center in accordance with the institution-approved animal care guidelines. All procedures were approved by the University of Perugia Animal Welfare Committee.

### 2.10. Surgical Technique

#### 2.10.1. Subcutaneous Transplant

The dorsum was prepared and scrubbed with povidone-iodine. A subcutaneous pocket was prepared on the dorsum. The DE was placed in the pocket, and the skin was sutured with 4-0 polyglactin interrupted suture.

#### 2.10.2. Full-Thickness Lesion Grafting

The dorsum was prepared and scrubbed with povidone-iodine. A circular full-thickness skin sample (1.5 cm diameter) was excised at the dorsal region. The DE or a cellular scaffold (S) was sutured on the back of the wound with 4-0 polyglactin. The surgical site was covered with secured tulle grease dressing. As a control, a circular full-thickness skin sample (1.5 cm diameter) was excised at the dorsal region. The wound was dressed with secured tulle grease, directly.

### 2.11. Statistical Analysis

For epidermis thickness measurements and for collagen evaluation, distributions of variables were assessed by the Shapiro-Wilk test and data were standardized, for each treatment, on mean and SD of normal skin thickness (*z*-score). Two-way analysis of variance for repeat measures (RM-ANOVA) was used to detect differences among the groups. A two-tailed *p* value < 0.05 was considered significant. qPCR data were expressed as mean ± standard deviation (SD) in at last three independent experiments. Statistical significance was determined by the calculation of the 95% CI.

Viability data were expressed as mean ± standard deviation (SD) in at last three independent experiments. Two-sided Student's *t*-test was used for comparison between groups. The significance level was set at *p* < 0.05. Statistical analyses were performed using IBM-SPSS version 21.0 (IBM Corp., Armonk, NY, USA, 2011).

## 3. Results

### 3.1. Development and Characterization of Dermal Equivalent (DE)

**Figure 1:**
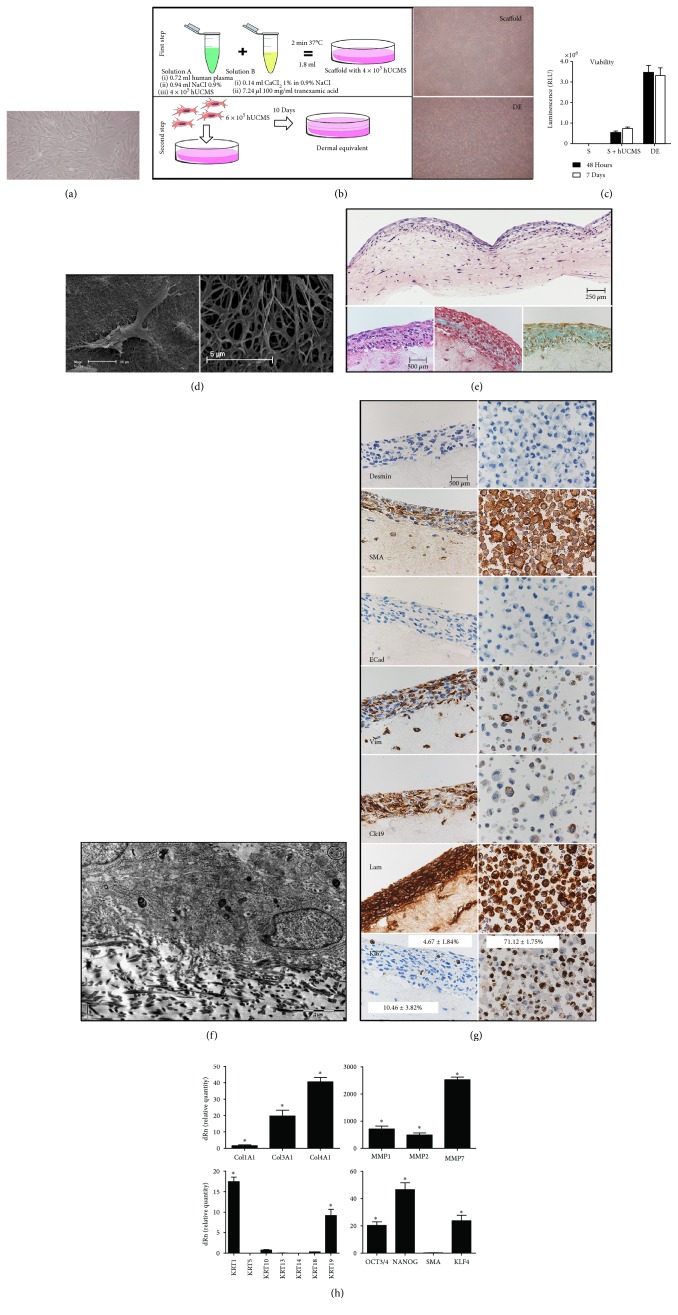
Construction and in vitro characterization of the dermal equivalent. (a) hUCMS morphological features in vitro. (b) Schematic representation of methods to constitute DE, with phase-contrast representative images of the scaffold (S) and DE. (c) The viability test, on the scaffold and DE at the indicated time in comparison with the cell-deprived fibrin scaffold, shows stable values at two different times. (d) Representative SEM images to illustrate the intimate connection between cell and fibrin and the 3D porous structure of fibrin itself. (e) Upper: representative histological H&E stain of the entire DE. Lower: magnification pictures of H&E staining, Masson staining, and keratin staining (left to right). (f) The representative TEM image to illustrate the superficial cell layer seeded on the fibrin scaffold; two cells are well identifiable and it is possible to see their cell membrane very closely. (g) Immunohistochemistry examination of DE in comparison with that of hUCMS maintained in culture conditions for the indicated markers. Ki67 positivity percentages in relation to superior cell multilayer or inferior entrapped cells, in comparison with the cell maintained in normal culture conditions. There is an evident decrease in Ki67-positive cells after DE construction (*p* < 0.001). (h) qPCR analysis for the indicated markers of DE in comparison with hUCMS alone was used as a calibrator. The expression of each marker, relative to its own control, is equal to 1. HPRT1 served for the control. All results appear as mean ± SD of three independent experiments (^∗^ *p* < 0.05).

hUCMS were prepared by our method [4] and expanded in vitro in CMRL supplemented with 10% fetal bovine serum (FBS) in polystyrene flasks that had been pretreated with hyaluronic acid (HA) (Figure 1(a)). Culture on HA allows for higher production of ECM as compared to untreated ones (data not shown). To generate DE, we use IV–VIII passaged cultured hUCMS. Initially, a fibrin scaffold is generated containing the cells; thereafter, upon O/N incubation, other cells are multiple layered on the scaffold (Figure 1(b)). Cell morphology was first assessed by phase-contrast microscopy. Fibrin matrix-entrapped cells appear spindle-shaped and homogeneously distributed throughout the scaffold (Figure 1(b)). Scanning electron microscopy (SEM) shows the cell embodied in fibrin plus the scaffold's texture, where polymerization creates a dense net holding the cells and allows for gas/nutrient diffusion (Figure 1(d)). H&E staining confirms homogeneous cell distribution within DE (Figure 1(e)). Cells embodied in the scaffold prevent its own degradation during culture maintenance. In fact, no cells containing a fibrin scaffold undergoes degradation in culture (data not shown). On the other hand, cells added subsequently do not penetrate the inner scaffold, but rather make contact on its surface where they form a dense layer (Figure 1(e)).

Cell viability keeps constant upon 7 days of culture (Figure 1(c)), as compared to 48 hours, as for both a simple scaffold and DE, indicating that cell populations have not undergone either apoptosis or excessive growth. TEM evidences that the outer layer of the multicellular construct seats on fibrin fibrils (Figure 1(f)). Two contiguous cells and their membranes make contact with no development of specialized junctures. Cell ultrastructure shows morphologically well-preserved endoplasmic reticulum (ER) indicating that DE surface cells are in good health and are metabolically active. Upon overlayering on a fibrin scaffold, the cells tend to form, during the days of culture, a multicellular construct. Here, at least three sections are identifiable: (a) the upper section was represented by flat-spindled cells; (b) the lower section was represented by round/polygonal-shaped cells, disorderly distributed; and (c) moreover, at the level of the two sections' border, an area comprised of few cells containing amorphous material was identified under trichrome staining as acidic mucopolysaccharides. Indeed, hUCMS lying on the DE surface form a cell multilayer expressing keratin by specific staining (Figure 1(e)). Immunohistochemistry on the DEs was used to assess if the 3D structure would affect cell differentiation as compared to that on cultured control cells (Figure 1(g)). hUCMS are positively stained for SMA (smooth muscle actin), vimentin, and CK19 that represent putative specific markers of these stem cells: positivity remains stable also when the cells form the DE (although not all DE surface cells keep positive for SMA and vimentin). Both in basal and within DE, hUCMS express laminin in excess; this ECM glycoprotein forms complexes with proteoglycans, collagens, and the other matrix components. However, neither hUCMS nor DE are positive for E-cadherin expression, a protein typical of epithelial cells, or for desmin, a typical marker of myoid cells indicating the induction of a muscle cell phenotype. We finally studied the expression of Ki67, a nuclear protein that is expressed by proliferating (in all the steps of the mitotic cycle) but not quiescent cells. Cells cultured on hyaluronic acid are positive for Ki67 (71.12%) while within the DE, positivity is 4.67% and 10.46% (outside and inside area, resp.). Hence, DE cells are likely associated with a decrease in proliferation capacity.

qPCR shows that the fibrin matrix clearly enhances cell mRNA expression for collagens 1, 3, and 4 as well as for metalloproteinases that remodel it (Figure 1(h)). Cytokeratin mRNAs, originally expressed by hUCMS, increase. KLF4 mRNA expression is induced within DE as compared to cultured cells. KLF4 is a negative regulator of the cell cycle, but it also plays an important role in cell differentiation during organogenesis of the skin, colon, and eye. SMA is a typical marker of myofibroblasts and hence of hUCMS; when these cells are organized within DE, the relative mRNA expression tends to decline. mRNA expression for transcription factors OCT4 and NANOG, implicated in the maintenance of cell pluripotency and self-renewal, is strongly upregulated in comparison with that in the cultured cells. qPCR and WB were used to assess if DE formation would interfere with cell immunomodulatory capacity (Figure S1A, B). In general, DE build-up increases the production of molecules that are associated with immunomodulation (Figure S1A, B).

### 3.2. Subcutaneous Transplant

**Figure 2:**
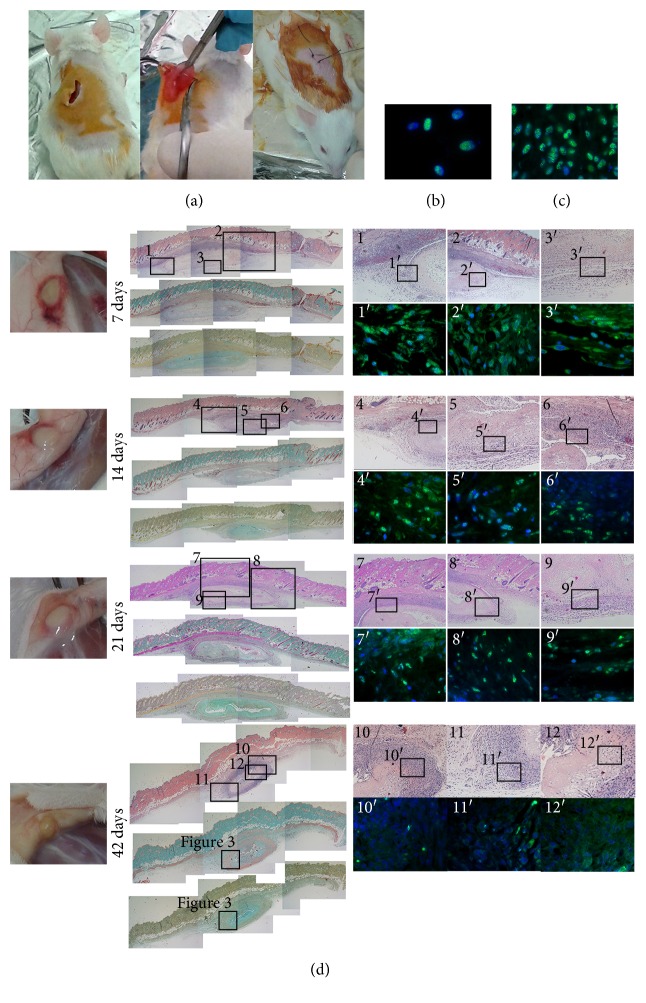
DE subcutaneous transplantation. (a) The method followed to insert DE subcutaneously. (b) The representative image of hUCMS maintained in normal culture conditions and staining with Ku80, a human nuclear-specific protein. The typical nuclear shape by staining of this protein is evident. (c) hUCMS in the scaffold preserve a clear positivity for Ku80 without environmental interferences. The appearance of cells and the scaffold stained with Ku80 is shown to fully appreciate the same analysis performed on DE. (d) Retrieval of DE at indicated times. DE is coated on the overlying skin in all animals treated and at all examined times. At the time of sacrifice, the skin graft area was overturned and this allows appreciation of DE well adherent to the overlying skin. In the implant area, it is not possible to detect signs of rejection or inflammation. On the contrary, an important vascularization at 7 days, fading away with the progress of the graft wound healing, appears. The DE is clearly visible at all times of the analysis. Representative images of the central sections of DE stained with H&E, Masson, and keratins. In any histological image of H&E, numbered rectangles indicate the respective magnification on the side. For some representative areas, immunofluorescence analysis for Ku80 showing the persistence of hUCMS up to 21 days is shown. In explants made at 42 days, positive Ku80 is very doubtful. In the sections of the central area of DE made with Masson staining or keratin staining, the squares indicate the areas that are presented enlarged in Figure 3. Tree mice were used for each time point.

To determine DE's full biocompatibility and immunocompetence, this has been grafted subcutaneously, in immunocompetent CD1 mouse (Figure 2(a)). After 7, 14, 21, and 42 d of Tx, the animals were exsanguinated for plasma collection and the graft was carefully removed for examination (Figure 2(d)). At any time points of graft, subcutaneous DE implant was devoid of any inflammatory reaction. At sacrifice, DE was clearly recognizable and coated on the overlying skin and not on the underlying musculature, while surrounding neovessel formation was intense at 7 days of Tx, thereby fading away (possibly related to surgery). No fibrotic tissue was evident at any time. DE showed time-related size reduction which may correlate to fibrin degradation. In parallel, Masson and peculiar histological staining showed a progressive increase in elastic fibers suggesting replacement of fibrin with collagen. The human nuclear protein Ku80 was used for labeling specifically hUCMS (Figures 2(b) and 2(c)); this marker evidenced that in the border area, hUCMS were mixed with unstained (possibly murine) cells (Figure 2(d)). By the time, it resulted progressively difficult to distinguish DE borders from the scaffold's central core being the only well-identifiable structure essentially comprised of few cells and large ECM. In explants made at 42 days, the Ku80 signal is lost.

**Figure 3:**
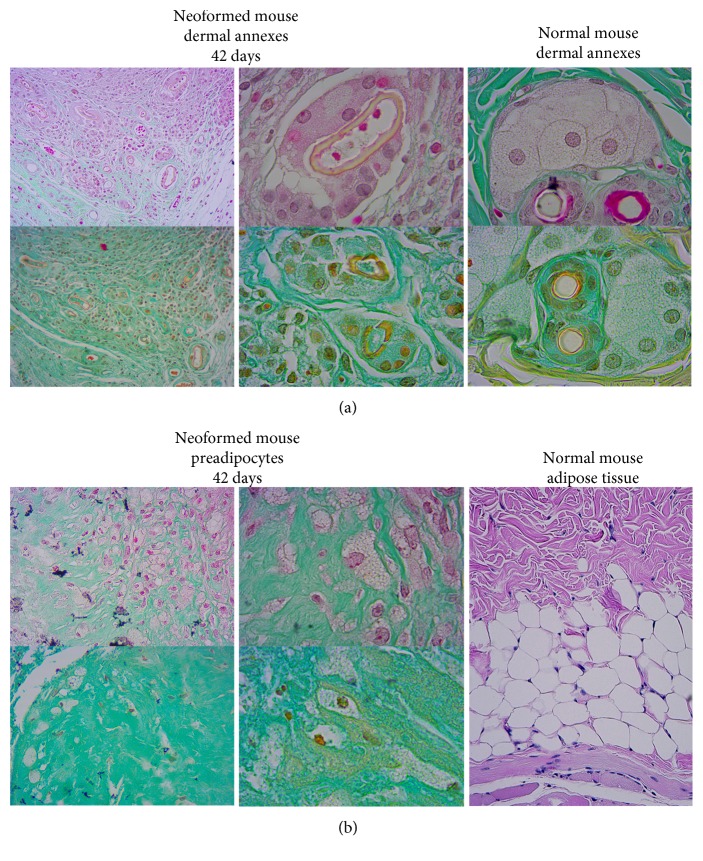
Neoformed mouse dermal annexes in DE explanted at 42 days. (a) The areas indicated in this figure, shown at higher magnification, highlight the presence of neoformation of many dermal annexes in comparison with annexes photographed in untreated skin areas of the same animals. The morphological similarity between the structures is evident, and peculiar is the area (below the hypodermis) in which these neoannexes are formed. (b) The central part of the DE, shown at higher magnification, highlights the presence of individual cells that resemble the morphology of preadipocytes; mature murine subcutaneous adipose tissue was shown for comparison.

Figure 3, DE at 42 d, depicts the extensive tissue neogenesis surrounding the remainder DE. Such tissue looks enriched in vessels and most importantly shows hair and subcutaneous gland budding. Within DE cells that are similar to preadipocytes, elements are detectable. These appear at 21 d of TX (data not shown). Therefore, at 42 d, the morphology of explants seems to indicate that DE enhanced the generation of murine dermal annexes.

CD3, CD11b, and CD20 immunophenotyping (Figure S2), within DE at different times, showed very weak presence of T and B cells or macrophages confirming the complete biocompatibility of the xenograft. Magnification of these sections showed the intimate association developed between murine tissues and the DE.

A reduction in IL1*β* and IL6, as assessed in the serum at sacrifice, throughout the study confirms that there is no rejection. Increased IL10 levels are associated with immune tolerance throughout 42 d as compared to those of controls (data not shown).

### 3.3. Full-Thickness Lesion Grafting

**Figure 4:**
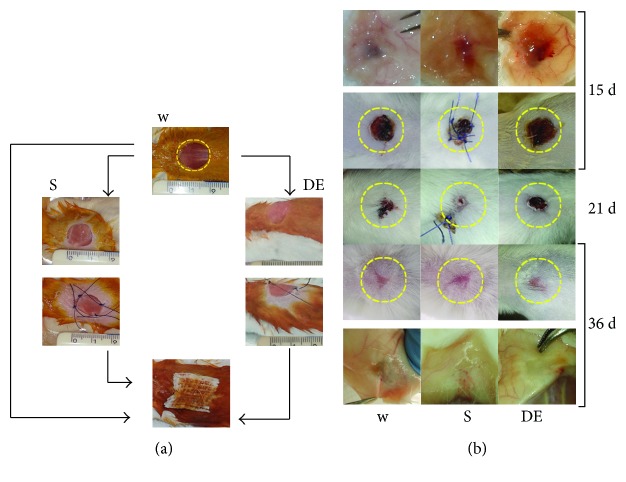
Full-thickness lesion DE grafting. (a) The technique used to implant DE or the scaffold (S) in a full-thickness lesion (W) at the level of the back of the mouse's neck. The original wound size (1.5 ± 0.1 cm) is indicated by the circle outlined in yellow. S and DE have a suitable resistance to easy suture it to the mouse skin; the same suture thread was used to hold in place the tulle grease medication. (b) Appearance of the lesion treated with the DE in comparison with the simple scaffold (S) or of the not treated wound (W) at three observation times, indicated in yellow, is the original diameter of the wound. The initial delay in the closure of the wound treated with DE is clear. For the observation points of 15 days and 36 days, the appearance of the lesion area on the contrary is also shown. The wound left untreated shows a very thin skin in comparison with the one treated with the DE to 36 days. Tree mice were used for each time point.

**Figure 5:**
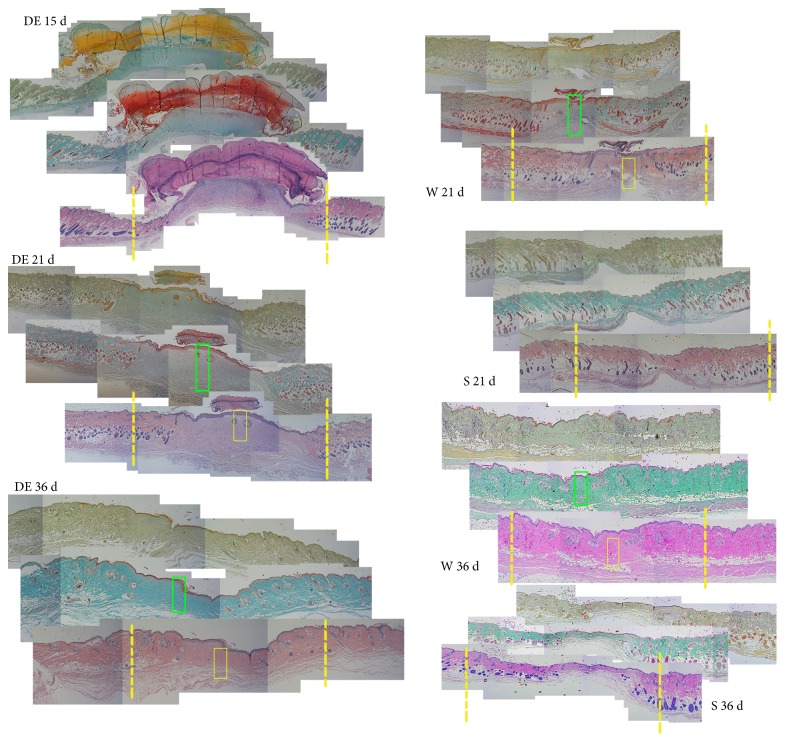
Morphological evaluation of the DE implant. DE histological analysis, at indicated times, on the central side by three different staining: H&E, Masson, and keratin, in comparison with that of the simple scaffold (S) or not treated wound (W). Of the image in H&E, the original extent of the wound is shown as a dashed yellow line. The green and yellow rectangles are the areas shown magnified in Figure 6.

Figure 4(a) indicates the graft method for DE implant in a full-thickness lesion at the level of the back of the mouse's neck. Lesion's diameter averaged 1.5 ± 0.1 cm. The wounds were treated with scaffold grafting (S) while the control group was treated with simple tulle grease medication (W). Grafted animals underwent explants at 15, 21, and 36 d. Wounds treated with DE seemed to heal slower than those treated with a scaffold only or sham, although the wound outcome looked much more better at the end (Figure 4(b)). During the healing process, the wound margins were regular and the thickness, as estimated by skin transparency at explant, was similar between the regenerated skin and the adjacent normal skin. No inflammatory reaction was observed (Figure 4(b)). The wound-containing skin was excised and histologically assessed. At different times, the central histological section, upon staining with H&E, Masson, or keratin, as compared with that of controls is shown in Figure 5. Scaffold graft (S) was associated with the generation of thin skin with no hypoderm (S21d and S36d).

**Figure 6:**
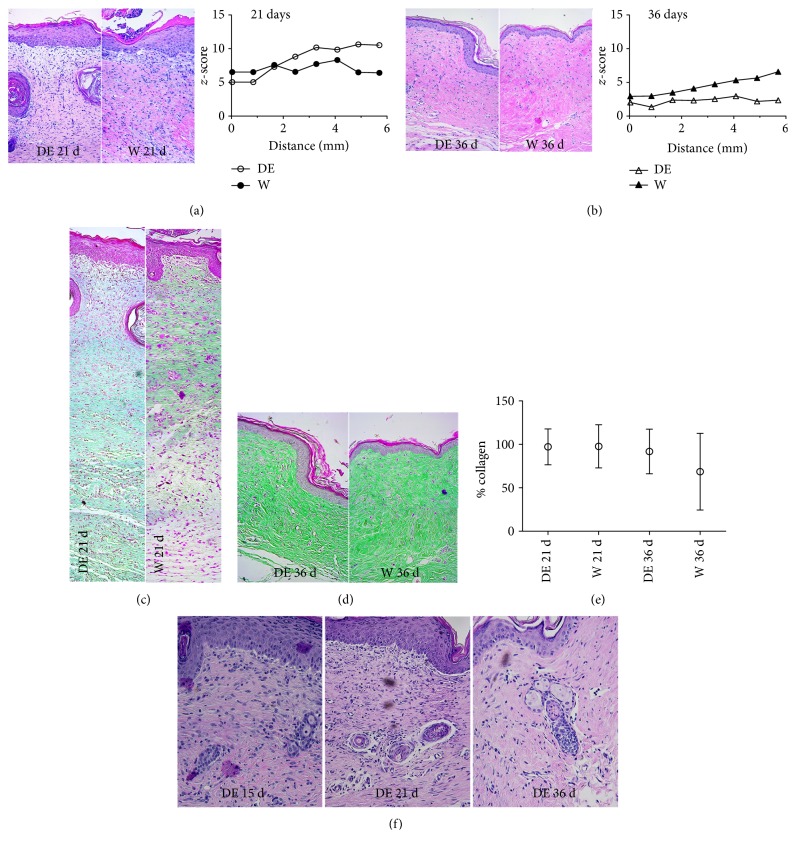
Full-skin restoration after the DE implant. (a, b) Magnification of H&E of the DE in comparison to that of the single wound at 21 and 36 days. The graph shows the values of epidermis thickness as a deviation from the normal value of the skin (*z*-scores), confirming the optimal healing of mouse epidermis treated with DE. *p* < 0.001 within subjects and between subjects at the two analyzed times. (c, d) Magnification of Masson's trichrome at 21 and 36 days to emphasize the presence and organization of the dermal collagen. (e) The average percentage of the collagen in the wound area and in the wound treated with DE at two times. *p* is not significant. (f) Representative magnifications of H&E to highlight the neogenesis of progressive dermal annexes in the center of the wound treated with ED at various times.

In particular, the DE showed plenty of granular tissue at 15, 21, and 36 days: in fact, DE allows for the regeneration of the derma, on a homogeneous fashion, which may have delayed healing. There was no retraction phenomenon otherwise visible in the wound (W21 and W36) as well as in the scaffold (S21 and S36). High-magnification examination in Figure 6(a) focuses on the wound central core upon treatment at 21 days as compared to the untreated wound after staining with H&E. Mouse dermis, after DE treatment, unlike that of the control, appeared to be very homogeneous with hair budding. At 36 days (Figure 6(b)), the wound was completely healed both in control and in DE recipient, although in the latter, homogeneous epidermis, in terms of thickness, and derma filled with neatly aligned elastic fibers were observed unlike those of controls.

Epidermis thickness, at the level of the half wound, in DE recipients, on consecutive slides, as compared to that in untreated controls, ranked as changes in mean healthy skin thickness values, are exhibited (*z*-score) (Figures 6(a) and 6(b)). The gap between healthy skin epidermis thickness and treated skin increases steadily by going into the wound core at 21 days in treated animals. Such a different trend indicates, in control animals, the presence of wound retraction that is absent in DE recipients (where only healing delay is present). At 36 days, changes in epidermis thickness (as compared to that in the healthy skin) are decreased and homogeneous across the entire DE wound treated. In control animals, thickness variations are constant and increase toward the wound center. This data may suggest that the healing process is different in the two animal groups: untreated wound heals rapidly but disorderly, due to wound retraction into scarring. On the contrary, DE-treated wound heals slowly, which allows for the regular assemblage of skin parts with no scarring. This is confirmed by high-magnification examination of slides stained with Masson staining (at the two reference times) that was consistent with that in collagen acidic fibers that were orderly assembled in DE (unlike control)-treated animals (Figures 6(c) and 6(d)).  Figure 6(e) also shows that the percentage of collagen in the wound is comparable between the two experimental animal groups: standard deviations confirm that the main difference between the two healing types is qualitative and not quantitative.  Figure 6(f) shows the high magnification, upon H&E staining, of the central wound area and evidences the formation of new dermal appendages at any time as expected.

## 4. Discussion

Wound healing is a complex process that requires the coordinated interplay of ECM, growth factors, and cells. Despite countless advances in biomaterial science in recent years, the majority of biomaterials employed for wound healing continue to be inadequate, due to difficulties in administration/delivery, poor biocompatibility, or other factors.

Here, we showed that a fibrin and mesenchymal adult stem cell epidermal equivalent was able to drive wound healing without scar formation. The employed DE is associated with several features: it contains fibrin derived from the same patient to whom the complex will be applied; hence, risk of adverse reactions and immune rejection will be minimal. Moreover, the polymerization process is controlled, by the modulation of both Ca^2+^ concentrations and temperature, to create scaffolds of the desired properties. The fibrin is associated with excellent hemostasis capacity, forming DE with good 3D configuration. In fact, our DE was highly porous and was interconnected with a clear 3D structure that traps the cells inside it but at the same time allows the free circulation of nutrients and gases. However, fibrin alone is associated with limited mechanical strength [22]. To overcome this obstacle, we have continued to culture DE in vitro for 10 days, which enabled the incorporated hUCMS to produce ECM bringing additional mechanical strength. These skills helped minimizing the phenomenon of skin stretching/contraction that leads to the formation of scars and keloids [20]. Recent reports showed that culturing macrophages on fibrin gels, fabricated by combining fibrinogen with thrombin-stimulated secretion of the anti-inflammatory cytokines, such as interleukin 10 (IL10). In contrast, exposure of macrophages to soluble fibrinogen stimulated high levels of the inflammatory cytokine tumor necrosis factor alpha (TNF-*α*) [23]. Hence, the DEs (per se) provide a valuable immunomodulatory support for tissue healing and regeneration. Furthermore, the presence of newly generated hyaluronic acid could increase the paracrine activity of hUCMS (discussed below) increasing their therapeutic effectiveness in wound healing [24].

Currently, most of the studies related to MSCs in wound skin clinical treatments are related to BM-MSCs; only limited studies mentioned the application of hUCMS [25, 26]. However, the process of obtaining hUCMS is much easier [4] and does not harm the donor, compared with the process of obtaining other mesenchymal cell types, with the possible exception of those retrieved from fat tissue. Nevertheless, while the use of autologous cells would possibly be better, in terms of either retrieval or culture maintenance or scaffold fabrication, still timing would be an obstacle. In fact, ulcers or lesions must be treated as soon as possible. Moreover, hUCMS are easy to freeze and thaw, which permits access to cell banking, within accurate microbiologic screening, offering a product that would be ready to be applied to the generation of epidermis equivalents. An additional interesting cell type, on this purpose, could be fetal fibroblasts. They were effectively applied to the treatment of diabetic wounds due to their immunoregulatory properties and cell matrix production [27, 28]. We believe that the latter report further strengthens the principle of using hUCMS: in fact, they share with fetal fibroblasts some properties, such as immunomodulatory activity, fetal origin, and a myofibroblast identity. hUCMS express cytoskeleton filaments that are muscle-specific in support of their “myofibroblast” features (a term developed by Majno et al. [29] to define cells that express, at ultrastructural level, common properties of smooth muscle cells and fibroblasts). In particular, contractile proteins such as desmin and alpha-actin of the smooth muscle (a marker of myofibroblasts), not muscular myosin, are expressed by these cells [4], while muscle myosin is absent. Furthermore, these cells also express vimentin [4], a protein of intermediate filaments that is of typical mesenchymal origin like fibroblasts, but vimentin is not expressed by smooth muscle cells. Coexpression of vimentin and desmin in these cells supports the idea that they intrinsically are myofibroblasts. Other interesting intermediate filaments expressed by hUCMS are cytokeratins that normally are expressed by epithelial cells of endodermic and ectodermic origin. Finally, the cells produce an extracellular matrix, like fibroblasts do.

hUCMS by their nature are low immunogenic and are able to mitigate the inflammatory environment of the wound by the secretion of various immunomodulatory molecules. In addition, hUCMS express molecules present at the maternal-fetal interface that are involved in the tolerogenic process: HLA-E, HLA-F, and HLA-G [15]. In particular, it has been described that the release of HLA-G can stimulate regulatory T cells, as also shown by us [10, 17].

We know that attenuation of the immune response is an important factor to ensure normal renovation of an injury while a continuous state of inflammation in the wound creates a cascade of events that perpetuates a nonhealing state. In addition, promotion of a tolerogenic environment results from the secretion of paracrine factors by hUCMS that could recall many cell types, including epithelial cells, endothelial cells, keratinocytes, and fibroblasts, and regulate a number of different cellular responses related to cell survival, proliferation, migration, and gene expression [30].

Further investigation has shown that dermal fibroblasts secreted increased amounts of collagen type I and alter gene expression in response to MSCs [31]. We think that in our case, this modulation depends on the ability of hUCMS to secrete ECM independently. In fact, the fibrin scaffold, during the days of in vitro culture, prior to transplantation in the animals, is filled with collagen and hyaluronic acid. We know that in the embryo, the ability of a wound to heal without a scar is due to a mitigated inflammatory environment [32] and to the ability to produce ECM with a different composition than that of adults [2, 33].

Our cells are very well suitable for scaffold creation; DE morphological characteristics thus obtained are reminiscent of those of normal human skin. In fact, hUCMS seeded on fibrin scaffolds remain on the surface where they organize to form a multilayered structure, as evidenced by staining with H&E.

In addition, through the Masson's trichrome and qPCR analysis, where there has been a sharp increase in the expression of collagen types III and IV and metalloproteases, we have shown that these cells are induced to synthesize large amounts of extracellular matrix (ECM).

Based on the qPCR data relative to the increase in Klf4 messenger associated with the decrease in the percentage of Ki-67 expression, to maintain viability, and the decline in the messenger for smooth muscle actin (SMA), we can assume that the DE cells have been induced to start an epithelial differentiation. In fact, the recent literature describes the increase in Klf4 necessary to epithelial differentiation because, among others, it stimulates the production of various types of cytokeratins of mature epithelia [34].

However, the obtained data indicate that the hUCMS are composed of multilayered and well-compacted cells, lacking well-recognizable layers of the differentiated skin. In addition, qPCR analysis shows the lack of expression of the messengers for cytokeratins 5 and 14 that are typical of the basal layers of the mature epidermis. Cells on hydrogels showed increased expression of transcription factors by hUCMS associated with the maintenance of pluripotency and self-renewal (Oct4, Nanog), as compared to cells grown in standard conditions.

Differentiation and paracrine signaling have both been implicated with mechanisms by which MSCs improve tissue repair. Our data show that at 42 days in the subcutis of the graft area, hUCMS completely disappeared (lack of Ku80 expression) but there was a marked neogenesis of murine dermal annexes as hairs and glands instead. So it is postulated that their action is effective at the level of paracrine signaling that regulates the local cellular responses to injury than at the level of a transdifferentiation [35]. Relevance associated with neogenesis of hair bulbs during the wound healing process, with respect to myoblast reprogramming (situated in the adipose tissue) with consequential minimization of scarring, due to regeneration of hypoderma, has been recently emphasized [36]. This is an interesting feature of our cells, because the conventional dermal substitute is not able to regenerate it. Furthermore, hair regeneration gives us the appearance of complete skin regeneration. Also, in the case of full-thickness wounds, thicker and homogeneous epidermis was obtained, as compared to that of controls. Possibly, hUCMS may stimulate murine resident stem cells (putatively murine myofibroblasts) of the skin and induce their proliferation and differentiation.

The best wound healing is shown in histological sections showing the elastic fibers after 36 days of transplantation as compared to the wound itself and the lack of scarring events.

## 5. Conclusions

A biomimetic scaffold consisting of hUCMS and fibrin (DE) is simple to fabricate, and it resembles the normal skin architecture. DE is highly biocompatible, stimulates regeneration of dermal annexes, and is able to enhance wound healing without scar formation.

These findings lead us to believe that the DE proposed model could represent a promising tool for the treatment of full-thickness epidermal injury, with foreseeable clinical applications, for instance, the treatment of diabetic foot ulcers.

## Supplementary Materials

Supplementary Materials Supplementary Table 1: primers used in qPCR. Figure S1: in vitro characterization of the dermal equivalent in comparison with the scaffold. Figure S2: CD3, CD11b, and CD20 DE cell immunophenotyping. Figure S3: full-thickness lesion DE grafting.
