# Supplementary material for: Human Umbilical Cord Wharton Jelly-Derived Adult Mesenchymal Stem Cells, in Biohybrid Scaffolds, for Experimental Skin Regeneration
Source: Stem Cells Int. 2017 Dec 31;2017:1472642. doi: 10.1155/2017/1472642 (PMC5804405; doi:10.1155/2017/1472642)
Supplement: Supplementary Materials — Supplementary Table 1: primers used in qPCR. Figure S1: in vitro characterization of the dermal equivalent in comparison with the scaffold. Figure S2: CD3, CD11b, and CD20 DE cell immunophenotyping. Figure S3: full-thickness lesion DE grafting. [file 1472642.f1.docx]

**Supplementary Table 1** : primers used in qPCR.

| ***Gene*** | ***T °C Annealing*** | ***Forward*** | ***Reverse*** | ***Expected lenght bp*** |
| --- | --- | --- | --- | --- |
|  |  |  |  |  |
| ABCG2 | 60 | 5’agggccaggagtccagtaat3’ | 5’tccaggagtggtcagattcc3’ | 211 |
| Col1a1 | 60 | 5’hhacacagaggtttcagtggt3’ | 5’gcaccatcatttccacgagc3’ | 185 |
| Col3a1 | 60 | 5’gctggcatcaaaggacatcg3’ | 5’gggagccctcagatcctctt3’ | 226 |
| Col4a1 | 60 | 5’ctgtggcaaatgtgactgcc3’ | 5’cggaggtcctcttgtccctt3’ | 184 |
| GAPDH | 60 | 5’gagtcaacggatttggtcgt3’ | 5’gacaagcttcccgttctcag3’ | 228 |
| HLAG1 | 60 | 5’gcccttgattgtgagattcc3' | 5’cttctccatcttcatctccac3’ | 138 |
| HLAG5 | 60 | 5'ctcaccttcacctcctttccca3' | 5'caatctgagctcttctttctccaca3' | 144 |
| HPRT1 | 60 | 5’ggtcaggcagtataatccaaag3’ | 5’ggactccagatgtttccaaac3’ | 250 |
| IDO1 | 60 | 5’gccttgcacgtctagttct3’ | 5’tcttggagagttggcagtaag3’ | 120 |
| IL10 | 60 | 5’gcctaacatgcttcgagatc3’ | 5’ggtcttcaggttctcccc3’ | 244 |
| IL17A | 60 | 5’ccataaccggaataccaatacc3’ | 5’ccagatcacagagggatatctc3’ | 118 |
| IL6 | 60 | 5’acagccactcacctcttc3’ | 5’cctcaaactccaaaagaccag3’ | 239 |
| iNOS | 60 | 5’gatgctgaactacgtcctg3’ | 5’ctgattttcctgtctctgtcg3’ | 215 |
| KFL4 | 60 | 5’cccacacaggtgagaaacct3’ | 5’atgtgtaaggcgaggtggtc3’ | 168 |
| Krt1 | 60 | 5’gattgccacctacaggaccc3’ | 5’gcttgtgctcacagacacac3’ | 91 |
| Krt10 | 60 | 5’gggaccaagatactaacaaaaccag3’ | 5’tggtctgtgtgaagggagact3’ | 139 |
| Krt13 | 60 | 5’tgagcatgaaagcggggc3’ | 5’gaggaagggaaaccaatcatcttg3’ | 250 |
| Krt14 | 60 | 5’agaccaaaggtcgctactgc3’ | 5’aggaggtcacatctctggatga3’ | 148 |
| Krt18 | 60 | 5’gagggctcagatcttcgcaa3’ | 5’ccagctgcagtcgtgtgata3’ | 191 |
| Krt19 | 60 | 5’cagtcacagctgagcatgaaag3’ | 5’gtagtgatcttcctgtccctcg3’ | 243 |
| Krt5 | 60 | 5’gaggaatgcagactcagtgga3’ | 5’ctgctacctccggcaagac3’ | 158 |
| MMP1 | 60 | 5’agtccagaaatacctggaaaaatac3’ | 5’ccacatcaggcactccacat3’ | 194 |
| MMP2 | 60 | 5’gctacgatggaggcgctaat3’ | 5’tcaggtattgcactgccaact3’ | 169 |
| MMP7 | 60 | 5’gtctctggacggcagctatg3’ | 5’gatagtcctgagcctgttccc3’ | 132 |
| NANOG | 60 | 5’caaaggcaaacaacccactt3’ | 5’tctgctgaaggctgaggtat3’ | 157 |
| OCT4A | 60 | 5’gagtcaacggatttggtcgt3’ | 5’gacaagcttcccgttctcag3’ | 152 |
| SOX2 | 60 | 5’catcacccacagcaaatgac3’ | 5’gcaaacttcctgcaaagctc3’ | 258 |
| TGFβ1 | 60 | 5’ggacaccaactattgcttcag3 | 5’cgggttatgctggttgtac3’ | 205 |
|  |  |  |  |  |





**Figure S1. In vitro characterization of Dermal Equivalent in comparison with Scaffold.**

**A)** In general, DE build-up increases production of molecules that are associated with immunomodulation. In particular, evident increase in HLAG5, TGFβ1, IL6, mRNA expression in the scaffold (S) 24h but mainly in DE (48h and 5d of production) in comparison with hUCMS is observed. HLAG1, iNOS, IL10, and IDO1 mRNAs, initially negative in cell monolayers, are present in S and DE at 48h but more a 5d of assemblage (Fig S1A). qPCR analysis for indicated markers of DE at two different time points in comparison with Scaffold and with hUCMS alone. Expression of each marker, relative to its own control, equals 1. HPRT1 served for control. All results appeared as mean ±SD of three independent experiments. *p<0.05. **B)** WB confirms the presence of HLAG and IDO1 protein in S 24h and in DE 5d (Fig S1B).

Representative immunoprecipitation analysis for IDO1 and HLAG in DE in comparison with Scaffold to ascertain presence of these proteins.

**Material and Methods: Western blotting**

Protein samples (40 μg) were analyzed on 10% or 12% (v/v) sodium dodecyl sulfate polyacrylamide gel electrophoresis and transferred onto a nitrocellulose membrane (Bio-Rad Laboratories). The employed antibodies were rabbit anti-human IDO1 (1:100; Thermo scientific, LiStarFish, Milan, Italy), rabbit anti-human iNOS (1:200; Thermo scientific,), mouse anti-human HLA-G (4H84) (1:200; Santa Cruz Biotechnology, Milan, Italy), and mouse anti-human Tubulin (1:8000; Sigma-Aldrich, Milan, Italy). Immuno-detection was performed by an Clarity™ Western ECL Substrate (Bio-Rad Laboratories) following vendor's recommendations.


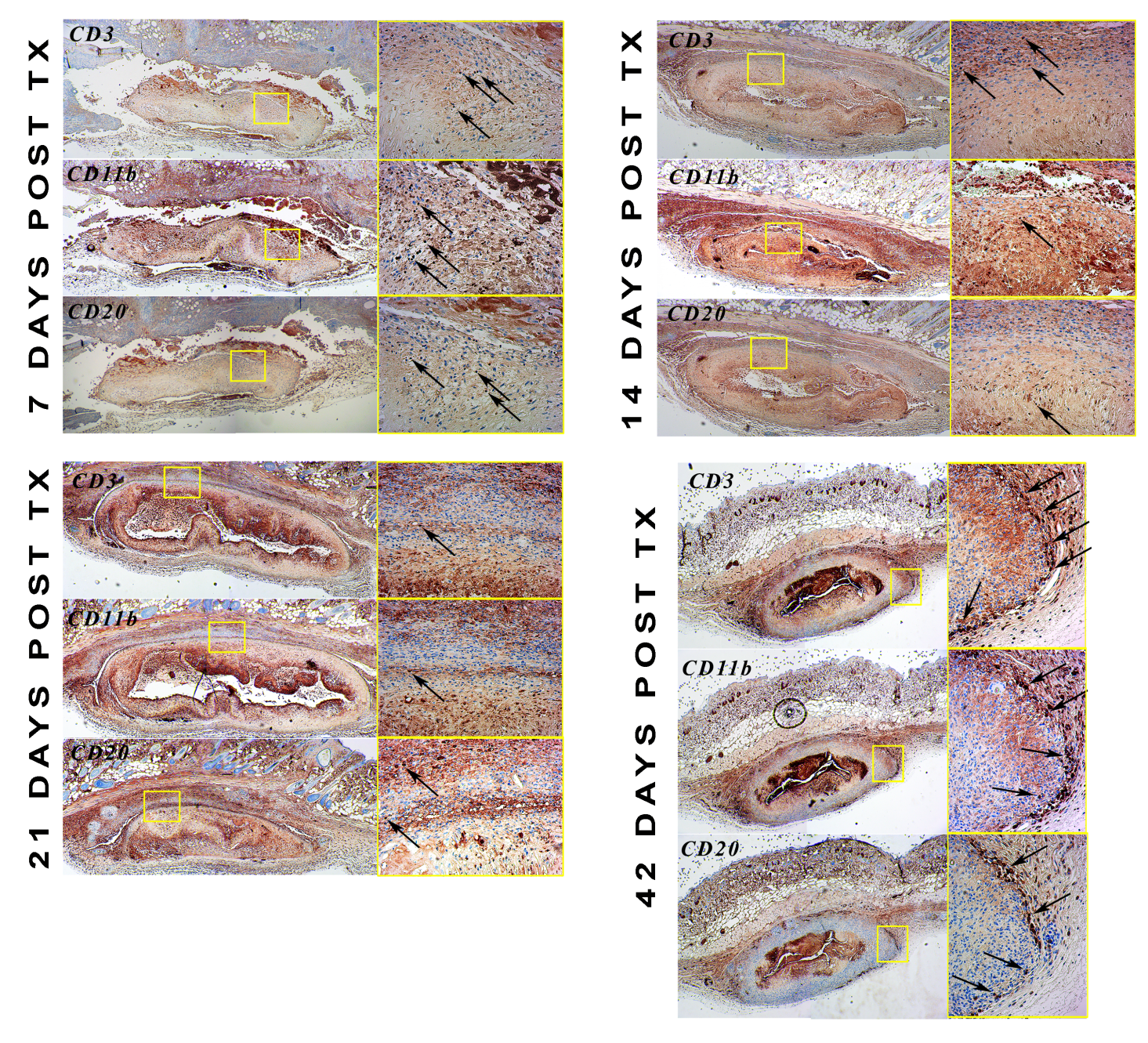


**Figure S2. CD3, CD11b, and CD20 DE cell immunophenotyping.**

CD3, CD11b, and CD20 immunophenotyping, within DE at different times, showed very weak presence of T and B cells or macrophages confirming the complete biocompatibility of the xenograft. Magnification of these sections showed the intimate association developed between murine tissues and the DE.


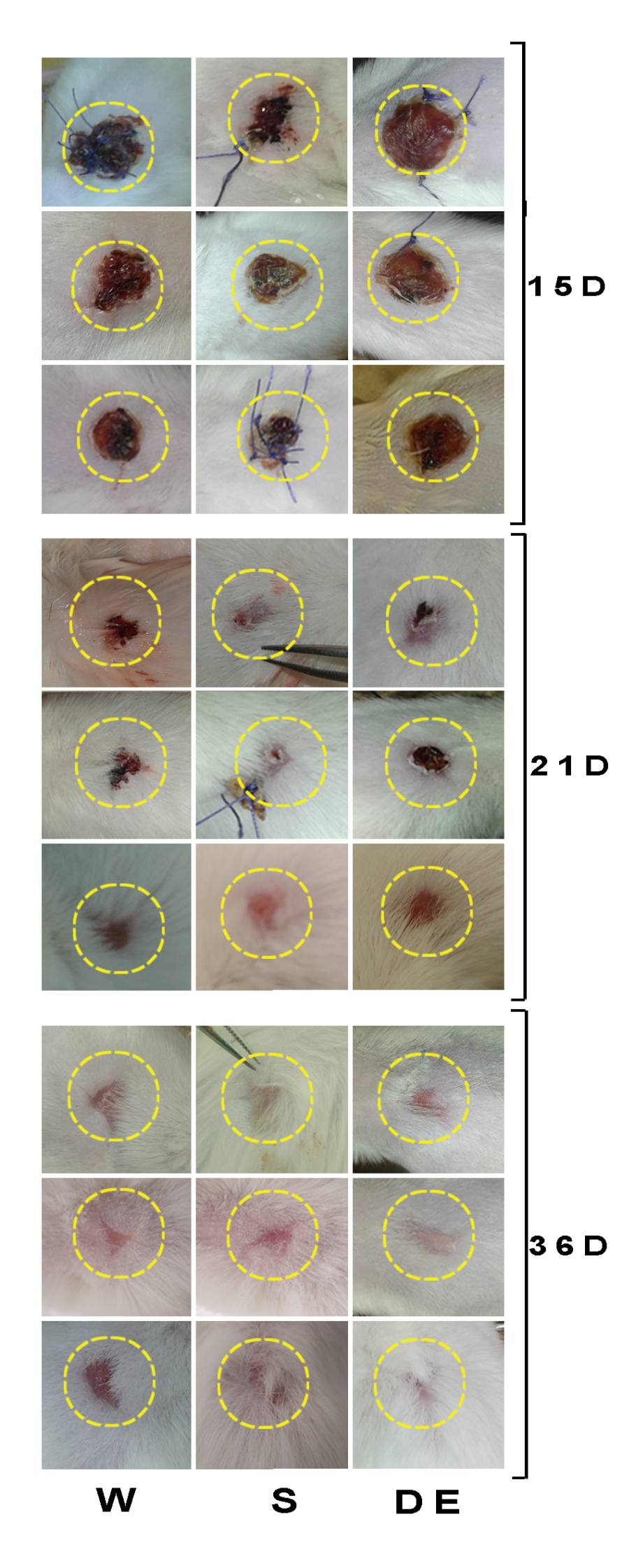


**Figure S3. Full-thickness lesion DE grafting.**

Appearance of the lesion treated with the DE in comparison with the simple scaffold (S) or not treated wound (W) at three observation times indicated, in yellow is the original diameter of the wound. It is clear the initial delay in the closure of the wound treated with DE. Three animals per experimental condition are shown.
